# OA09 Too few platelets, too many clots

**DOI:** 10.1093/rap/rkag113.009

**Published:** 2026-11-03

**Authors:** Sajeel Ahmed, Kenzie Maginnis, Rebecca Batten

**Affiliations:** South Tyneside and Sunderland NHS Foundation Trust, Sunderland, United Kingdom; South Tyneside and Sunderland NHS Foundation Trust, Sunderland, United Kingdom; South Tyneside and Sunderland NHS Foundation Trust, Sunderland, United Kingdom

## Abstract

**Introduction:**

Antiphospholipid syndrome (APS) is a systemic autoimmune disease characterised by arterial and venous thrombosis. The 2023 ACR/EULAR classification criteria recognise thrombocytopenia as a haematological manifestation of APS; however, severe thrombocytopenia remains uncommon and may be mistaken for primary immune thrombocytopenia (ITP). We describe a 51-year-old woman with established APS who developed profound thrombocytopenia that was initially managed as refractory ITP, resulting in interruption of anticoagulation and subsequent catastrophic arterial and venous thrombosis. This case highlights the importance of recognising thrombocytopenia as active APS requiring immunomodulatory therapy rather than modification of anticoagulation alone.

**Case description:**

A 51-year-old woman with APS diagnosed following an ischaemic stroke at the age of 20, leaving residual left-sided hemiparesis, and a subsequent deep vein thrombosis, had been maintained on long-term warfarin but was no longer under rheumatology follow-up. In August 2022, routine blood tests demonstrated severe isolated thrombocytopenia (platelet count 21 × 10⁹/L). She remained asymptomatic without petechiae, bruising or mucosal bleeding.

Repeat antiphospholipid antibody testing confirmed persistently high-titre anticardiolipin IgM (>472) and anti-β2 glycoprotein I IgM (420) antibodies, together with ANA (1:320 speckled) and anti-Ro positivity. However, dsDSNA, complements levels and clinical assessment did not support concomitant systemic lupus erythematosus. Initial management by haematology favoured refractory immune thrombocytopenia. She received repeated courses of corticosteroids with only transient improvement, followed by eltrombopag and mycophenolate, but platelet counts remained persistently low.

Warfarin was discontinued because of concerns regarding bleeding risk in context of worsening thrombocytopenia (platelets 4× 10⁹/L). Two months later she presented with critical limb ischaemia. Imaging demonstrated a 10 cm occlusion of the left superficial femoral artery with extensive ipsilateral venous thrombosis, requiring intravenous followed by subcutaneous heparin before recommencement of warfarin.

Rheumatology review recognised severe thrombocytopenia as a manifestation of active APS rather than isolated ITP. Further assessment, including bloods and cross sectional imaging, identified chronic kidney disease (eGFR 30 mL/min/1.73 m²) likely secondary to APS microangiopathy and autosplenectomy secondary to previous splenic infarction. Treatment was directed at active APS with intravenous and oral corticosteroids, hydroxychloroquine and rituximab alongside continued anticoagulation. Platelet counts subsequently normalised and have remained stable, allowing maintenance warfarin (target INR 2–3). She remains clinically well on maintenance prednisolone, hydroxychloroquine and 9-monthly rituximab with no further thrombotic events.

**Discussion:**

This case illustrates several important but under-recognised aspects of antiphospholipid syndrome. Although thrombocytopenia is recognised within the 2023 ACR/EULAR APS classification criteria, it is typically mild. Severe immune thrombocytopenia requiring multiple immunomodulatory therapies is uncommon and may obscure the underlying thrombotic nature of the disease.

The principal learning point was recognising thrombocytopenia as a manifestation of active APS rather than simply a bleeding disorder. Management initially focused on the low platelet count, leading to withdrawal of anticoagulation. However, within two months the patient developed recurrent arterial and venous thrombosis, emphasising that profound thrombocytopenia does not protect against thrombosis in patients with high-risk APS.

This case also highlights the multisystem nature of APS. In addition to previous arterial and venous thrombosis, the patient developed severe immune thrombocytopenia, progressive chronic kidney disease suggestive of APS nephropathy and autosplenectomy secondary to thrombotic disease. These manifestations demonstrate that APS extends beyond recurrent thrombosis and requires a holistic assessment of disease activity and organ involvement.

Successful treatment required control of the underlying autoimmune disease rather than thrombocytopenia alone. Prednisolone, hydroxychloroquine and rituximab resulted in sustained platelet recovery, allowing safe continuation of life-long anticoagulation and preventing further thrombotic events. This supports the concept that immunomodulatory therapy may be essential in selected patients with APS complicated by severe thrombocytopenia.

This case raises an important question for discussion: in patients with high-risk APS and severe immune thrombocytopenia, should management focus primarily on controlling immune-mediated disease activity to permit continuation of anticoagulation, rather than modifying anticoagulation alone?

**Key learning points:**

• This case reinforced that severe thrombocytopenia in antiphospholipid syndrome should not be interpreted solely as a bleeding risk. In patients with previous arterial or venous thrombosis, profound thrombocytopenia may represent active immune-mediated APS occurring alongside persistent thrombotic risk.

• A major learning point was recognising that thrombocytopenia is now incorporated within the 2023 ACR/EULAR APS classification criteria, although severe thrombocytopenia requiring intensive immunomodulatory therapy remains uncommon. The coexistence of profound thrombocytopenia and recurrent thrombosis presents a challenging therapeutic dilemma that requires careful balancing of bleeding and thrombotic risks.

• The case also highlighted the importance of treating the underlying autoimmune disease. Withdrawal of anticoagulation because of persistent thrombocytopenia was followed by recurrent thrombosis, whereas treatment with prednisolone, hydroxychloroquine and rituximab normalised platelet counts and enabled safe continuation of long-term anticoagulation. This illustrates that immunomodulatory therapy may be required not only to correct thrombocytopenia but also to reduce thrombotic risk by controlling APS disease activity.

• The principal learning objective from the conference would be to better understand optimal management strategies for patients with high-risk APS complicated by severe immune thrombocytopenia. I would welcome discussion regarding platelet thresholds for anticoagulation, indications for immunomodulatory therapy including rituximab and hydroxychloroquine, and the management of renal involvement suggestive of APS nephropathy.

